# Evaluating ‘Power 4 a Healthy Pregnancy’ (P4HP) – protocol for a cluster randomized controlled trial and process evaluation to empower pregnant women towards improved diet quality

**DOI:** 10.1186/s12889-022-12543-z

**Published:** 2022-01-21

**Authors:** Renske M. van Lonkhuijzen, Susanne Cremers, Jeanne H. M. de Vries, Edith J. M. Feskens, Annemarie Wagemakers

**Affiliations:** 1 Health and Society, Department of Social Sciences, Wageningen University & Research, Hollandseweg 1, bode 60, 6706KN Wageningen, The Netherlands; 2 Human Nutrition & Health, Department of Agrotechnology and Food Sciences, Wageningen University & Research, Stippeneng 4, bode 62, 6708WE Wageningen, The Netherlands

**Keywords:** Pregnant women, Pregnancy, Midwifery, Empowerment, Diet quality, Nutrition, Health promotion, Multidisciplinary collaboration

## Abstract

**Background:**

In general during pregnancy, women are aware of the importance of good diet quality, interested in nutrition, and receptive to changing dietary intake. However, adherence to dietary guidelines is sub-optimal. A pregnant woman’s first information source regarding nutrition information is her midwife. Healthy nutrition promotion by midwives may therefore be very promising, but midwives face multiple barriers in providing nutritional support. Empowering pregnant women to improve their diet quality is expected to improve their health. Therefore an empowerment intervention has been developed to improve diet quality among pregnant women. The objective of this study is to evaluate the effectiveness and feasibility of Power 4 a Healthy Pregnancy (P4HP). P4HP aims to empower pregnant women to have a healthier diet quality.

**Methods/design:**

This study applies a mixed methodology consisting of a non-blinded cluster randomized trial with an intervention (P4HP) group and a control group and a process evaluation. Midwifery practices, the clusters, will be randomly allocated to the intervention arm (*n* = 7) and control arm (*n* = 7). Participating women are placed in intervention or control conditions based on their midwifery practice. Each midwifery practice includes 25 pregnant women, making 350 participants in total. Health related outcomes, diet quality, empowerment, Sense of Coherence, Quality of Life, and Self-Rated Health of participants will be assessed before (T0) and after (T1) the intervention. The process evaluation focuses on multidisciplinary collaboration, facilitators, and barriers, and consists of in-depth interviews with midwives, dieticians and pregnant women.

**Discussion:**

This study is the first to evaluate an empowerment intervention to improve diet quality in this target population. This mixed method evaluation will contribute to knowledge about the effectiveness and feasibility regarding diet quality, empowerment, health-related outcomes, multidisciplinary collaboration, facilitators and barriers of the empowerment intervention P4HP. Results will help inform how to empower pregnant women to achieve improved diet quality by midwives and dieticians. If proven effective, P4HP has the potential to be implemented nationally and scaled up to a long-term trajectory from preconception to the postnatal phase.

**Trial registration:**

The trial is prospectively registered at the Netherlands Trial Register (NL9551). Date registered: 19/05/2021.

**Supplementary Information:**

The online version contains supplementary material available at 10.1186/s12889-022-12543-z.

## Background

A healthy diet is important for everyone, but crucial during pregnancy for the health of both mother and child [1–5]. During pregnancy, women are aware of the importance of a good diet quality and are interested in nutrition [6, 7]. However, adherence to dietary guidelines and recommendations is sub-optimal, especially among pregnant women with lower socioeconomic status (SES) [8–10], and specifically for the intake of fruit, vegetables, grains, folate, and iron [11–15]. Poor diet quality by the mother is associated with adverse health outcomes, including increased risk of pre-eclampsia, gestational diabetes, and excessive gestational weight gain. For the unborn, a poor diet of the mother is related to adverse birth outcomes, including premature birth and low birth weight, as well as disadvantageous health outcomes later in life, such as the increased risk of developing chronic diseases [1–3]. However, due to several challenges, such as nausea, cravings, and ingrained habits, pregnant women experience difficulties with the implementation of dietary changes and sustaining these changes during their pregnancy [8, 16]. On top of that, the diet quality of pregnant women is challenged by aspects such as the costs of living and their physical and social environments.

Pregnancy is often regarded as a critical transition, a teachable moment, in which women are more receptive to changing dietary patterns than in other phases in life [6, 7, 17–19]. Pregnancy might increase awareness regarding diet since women generally feel that one of the few things to positively impact the health of their child is to make dietary improvements [20]. A pregnant woman’s first, most important, and most trusted information source regarding nutrition information is their midwife [6, 7, 21–25]. Also, midwives feel responsible to inform pregnant women about a healthy diet [25]. Compared to other countries, midwives in the Netherlands play a relatively large and central role in maternity care. Healthy nutrition promotion by midwives is therefore promising to make use of this window of opportunity where women are increasingly aware of their behaviours for their health and their child’s health [21, 23, 25, 26]. However, although midwives feel the responsibility to provide nutritional advice, they do not consider themselves nutritional experts and encounter structural barriers in providing nutrition communication [5, 25, 27–29]. Some of the main barriers for midwives are time constraints and unsupportive health systems (e.g. a lack of cooperation with other health professionals) [5, 29–32], as well as limited relevant and reliable resources and training [5, 29, 33–35]. As a result, nutrition communication in antenatal care generally remains suboptimal. Currently, only Dutch women with pregnancy complications, overweight/obesity, or excessive gestational weight gain, or those who asked for it themselves receive comprehensive guidance regarding their nutrition during pregnancy [25, 36, 37].

The modern concept of empowerment is increasingly defined as strengthening the capabilities and the self-reliance of individuals [38]. We use Aubel’s definition, who describes empowerment as ‘the ability of individuals or groups to improve capacities, to critically analyse situations and to take actions to improve those situations’ [39]. This definition applies bottom-up thinking to drive behaviour change [40–42], requiring an environment in which pregnant women can engage in open communication [40]. The process of empowering pregnant women is expected to improve their health, as they are supported to make healthier choices, for example in terms of nutrition [6, 21]. This can therefore result in improved child health and providing children with a healthy and successful start of life [2, 3].

P4HP has been developed in the past years together with stakeholders, aiming to empower pregnant women to have a healthier diet quality [42–45]. This intervention may contribute to enduring new-borns with a healthy, successful start of life [2, 3] and has the potential to improve health across generations. P4HP uses a women-centered empowerment approach, to prioritize the woman’s individual needs, as defined by the woman herself, assigning to the woman’s choice, control, and continuity of care. P4HP allows women to be empowered, gaining control over their lives and learning how to achieve goals that are meaningful to them. Individuals are most likely to change their behaviour to make healthier choices when they are educated and motivated to do so, in addition to environments and policies supporting these decisions [46].

A similar intervention in which empowerment and diet quality among pregnant women is central has not been implemented before to our best knowledge. The research will contribute to theoretical development by providing practice-based evidence [47, 48]. This mixed methods study aims to 1) evaluate the effectiveness of P4HP regarding diet quality, empowerment, Sense of Coherence (SOC), Quality of Life (QoL), and Self-Rated Health (SRH) using a cluster randomized controlled trial (C-RCT), and 2) evaluate P4HP in terms of multidisciplinary collaboration, facilitators and barriers using a process evaluation. This way, as we retrieve both information about what is needed to achieve an effect and about what is needed in the implementation and in the multisectoral collaboration, we gain insight in both the effectiveness as well as the feasibility of P4HP.

**Fig. 1:**
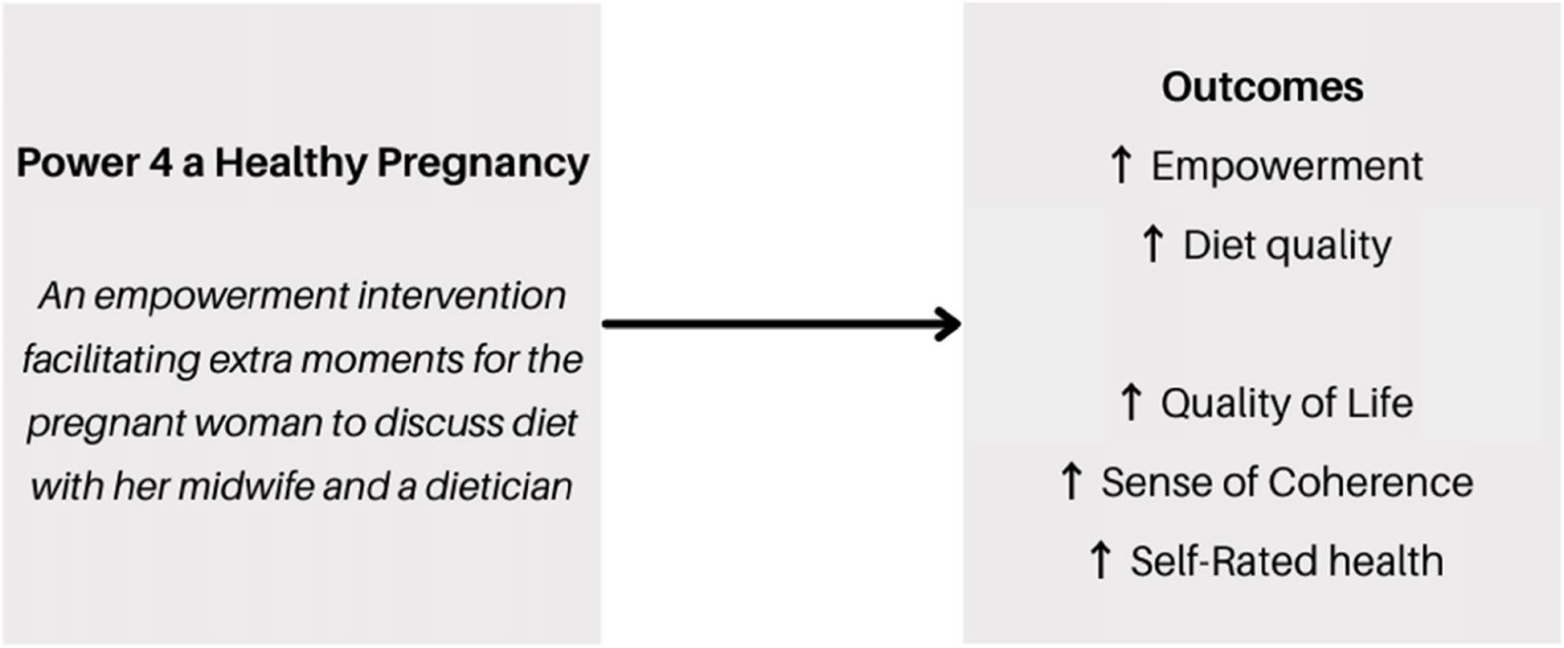
Overview of hypothesis P4HP

Research in the area of empowerment towards dietary intake in pregnancy is sparse [24, 49]. Still, empowerment has been linked to diet quality, although mostly in global south [50–53]. Additional to empowerment and diet quality, health outcomes are included, as empowerment has been previously linked to the concepts QoL [54, 55], SOC [56, 57], and SRH [58, 59]. Also diet quality has been linked to the concepts QoL [60, 61], SOC [62, 63], and SRH [64, 65]. We hypothesize that empowerment, improved diet quality, and the health outcomes QoL, SOC and SRH will have a mutually reinforcing, invigorative effect on each other (Fig. 1).

## Methods

### Study design

This mixed methods study consists of a non-blinded C-RCT with an intervention (P4HP) group and a control group and a process evaluation. A qualitative process evaluation will take place to evaluate P4HP by midwives and dieticians in terms of multidisciplinary collaboration, facilitators, and barriers.

A pilot study has launched on October 1st, 2021, with the aim to reach a total of 10 participants from two midwifery practices within 2 months. In this pilot study we pre-test the questionnaires, the perceptions of the P4HP intervention, and the practical and technical matters regarding implementation, including the organization of multidisciplinary collaboration. Any suggestions by the midwives, dieticians, and pregnant women will be duly accommodated to improve the feasibility.

### Non-blinded C-RCT

**Fig. 2:**
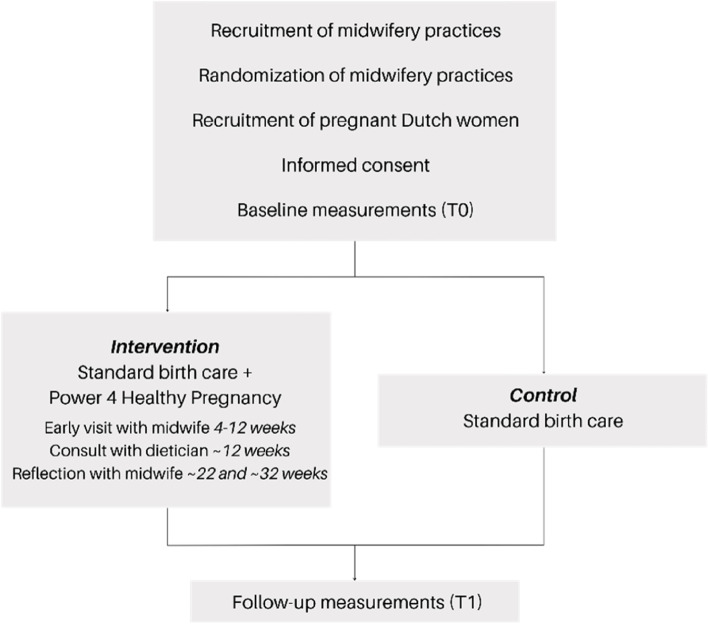
Flow diagram of the participants through the trial

The non-blinded C-RCT will evaluate the effectiveness of P4HP on pregnant women’s empowerment, diet quality, and health outcomes between intervention and control practices. Figure 2 details the flow of participants from recruitment of midwifery practices until the last follow-up contact for intervention and control participants. The clusters are midwifery practices in the Netherlands and the participants are Dutch-speaking pregnant women visiting the practice. Cluster randomization is applied to eliminate the risk of cross-contamination between the two study arms. Thus, whether pregnant women are placed in the intervention or the control group is based on whether receiving care from intervention or control midwifery practices. Due to the nature of the intervention, it is not possible to blind the professionals, participants, or investigators to the study conditions. This protocol has been written according to the recommendations of the Standard Protocol Items Recommendations for Interventional Trials (SPIRIT) 2013 statement [66, 67] (Additional file 1). SPIRIT guides key content to facilitating the drafting of high-quality protocols, including recommendations for intervention trials.

### Cluster and participant recruitment

Midwifery practices in the Netherlands will be recruited by using existing connections, snowballing, social media, and presentations of the study at local collaborations of midwifery practices. Midwifery practices are randomized as clusters to either an intervention arm or standard birth care. Clusters will be randomized by a researcher, who is unfamiliar with the midwifery practices, using a randomization scheme in Excel.

Eligible pregnant women will be recruited in randomized midwifery practices (clusters) in the Netherlands using a purposive sampling technique. Midwives will be informed by the research team about the in- and exclusion criteria. Midwives will explore whether or not the pregnant women meet the inclusion criteria and explain to them the purpose of the study. The women will be invited to participate voluntarily. To be eligible to participate in this study, a participant must meet all of the following criteria: being in the first trimester of pregnancy; > 18 years of age; understanding and speaking Dutch; consuming a Dutch diet pattern i.e. a diet with a maximum of one hot meal per day. A potential participant who meets any of the following criteria will be excluded from participation: not willing to provide informed consent; having a severe chronic illness/condition (for example cancer); having conditions that may affect diet quality. The recruitment of participants will begin on 01-01-2022 and will end when all 14 midwifery practices have met their target of 25 women. Alternatively, the recruitment will end on 31-12-2022.

### Sample size estimation

The sample size estimation is based on the design of a C-RCT [68] using the Group-or Cluster-Randomized Trials sample size calculator from the National Institutes of Health [69], where each cluster represents a midwifery practice. The results indicate that to detect an effect size of 0.4 (small to medium) [70] with a power of 80%, an alpha of 0.05, and an intra-cluster correlation coefficient of 0.02, each arm should include 7 clusters of 25 participants each. The assumption of an intra-cluster correlation coefficient of 0.02 is based on our experience with the SLIMMER project, a cluster randomized trial of a combined lifestyle intervention including behaviour change in diet and physical activity on overweight and risk of diabetes [71]. We strive to keep the number of participating pregnant women per cluster as similar as possible. Based on the sensitivity analysis for this sample size estimation [69], we estimate that 25 participants from 7 intervention and 7 control clusters – leading to 350 participants in total – will be sufficient to detect the relevant difference between groups. The Netherlands had 168.066 births in the year 2020 [72]. Three hundred fifty participants represent < 0.25% of pregnancies in the Netherlands, and therefore expected to be achievable.

### Data collection and assessments

**Fig. 3:**
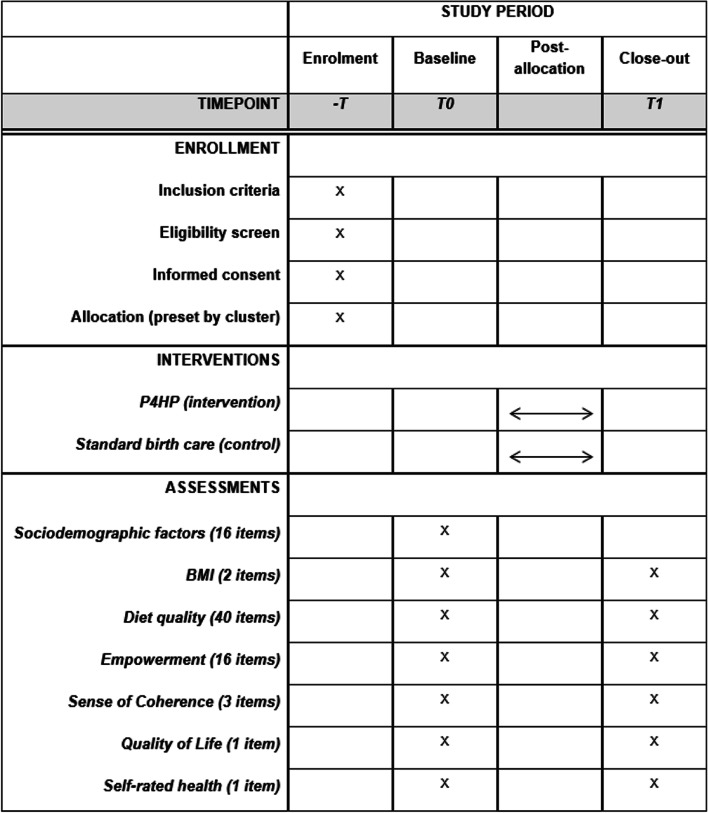
P4HP C-CRT SPIRIT diagram

At enrolment, participants will give their informed consent through consent forms. Subsequently, participants will be provided with two online quantitative questionnaires to fill out, utilizing baseline data for the study (T0). Upon completion of P4HP, participants are provided the same two online quantitative questionnaires (minus sociodemographic data) to respond to the post-intervention assessment (T1) (Fig. 3). The first questionnaire assesses diet quality [73]. The term diet quality has been used in recent decades to evaluate the dietary habits or patterns of a population and the efficacy of dietary interventions [74–76]. Diet quality is a suitable term to present multiple food components, assessed using an index to evaluate the extent of adherence to dietary guidelines. The second questionnaire includes all other assessments and is distributed using Qualtrics. Access to all data collection tools and databases is strictly limited and regulated through personal user profiles. Both platforms are password-protected and all data will be regularly backed up into a password-protected database.

#### Sociodemographic data

Questions to collect name, year of birth, phone number, email, postal code digits, living situation, ethnicity, educational level, and personal and household income.

#### BMI

Two questions to collect height and body weight.

#### Diet quality

We derive scores for diet quality using Eetscore [77]. Eetscore is a validated web-based screening tool to determine the diet quality of Dutch adults and suitable for assessing change in diet quality over time. Eetscore consists of a short food frequency questionnaire in an accessible writing style that is scored with the Dutch Healthy Diet index 2015 (DHD2015-index) to assess adherence to the Dutch food based dietary guidelines of 2015 of the Dutch health Council [73]. The DHD2015-index has been validated with 24 h dietary recall and FFQ data. In addition, to the DHD2015-index, a 16th component for unhealthy choices has been added. The questionnaire to determine this diet quality score consists of 40 questions with sub-questions inquiring about the consumption of 54 foods or food groups. Based on the answers a total score and 16 sub scores will be calculated. Sub scores of Eetscore are available for 1) vegetables, 2) fruit, 3) whole grain products, 4) legumes, 5) nuts, 6) dairy, 7) fish, 8) tea, 9) fats and oils, 10) coffee, 11) red meat, 12) processed meat, 13) sugar-containing beverages, 14) alcohol, 15) sodium and 16) unhealthy choices. Each component will be scored on a scale ranging from 0 (non-adherence) to 10 (complete adherence), providing a total score between 0 and 160. For the calculation of the scores, specific cut-off and threshold values are used. Since Eetscore is developed for the general Dutch adult population, it has been adapted to fit the requirements of pregnant women. The (sub)scores were adapted based on the dietary recommendations for pregnant women provided by the Dutch Health Council and the Netherlands Nutrition Centre [78]. We use the web-based version of Eetscore which can be filled out in about 10-15 min.

#### Empowerment

Empowerment will be assessed using the Pregnancy-Related Empowerment Scale (PRES). This is a valid and reliable assessment containing 16 questions on women’s health-related empowerment during pregnancy [79].

#### SOC

We derive SOC scores from the three-item SOC questionnaire (SOC-3) [80, 81]. This validated questionnaire includes three questions; all corresponding to the three components of SOC: comprehensibility (“Do you usually feel that the things that happen to you in your daily life are hard to understand?”), manageability (“Do you usually see solutions to problems and difficulties that other people find hopeless?”), and meaningfulness (“Do you usually feel that your daily life is a source of personal satisfaction?”) [80–83]. Participants can indicate their answer as 1 (yes, usually), 2 (yes, sometimes), or 3 (no). The sum of these three items (after reverse coding of the comprehensibility item) reflects the total SOC, with higher scores indicating a weaker SOC. Participants are divided into three SOC groups: weak (scores 6–9), intermediate (scores 4–5), and strong (score 3) - in line with previous studies [80, 84, 85]. We use a Dutch version of the SOC-3, a short version of the original to alternatively measure change, as previously used by Herens [85–87]. SOC can be influenced by interventions and has been previously linked to the concept of empowerment [56, 57].

#### QoL

We derive a score for global QoL using a Visual Analogue Scale (VAS): a horizontal line of 100 mm, with stops (“anchors”) at both extremes – 0 (worst imaginable QoL) to 100 (perfect QoL) – representing the limits of self-rated QoL. A QoL VAS is a frequently used single-item technique with good to excellent levels of reliability, validity, and sensitivity [88–90].

#### SRH

We derive a score for SRH using a General Self-Rated Health (GSRH) question. Asking people to rate their health in surveys provides an easily assessed, common indicator of health [91–95]. Respondents are asked to rate their health, in general, as ‘excellent’, ‘very good’, ‘good’, ‘fair’ or ‘poor’.

### Process evaluation

To perform a process evaluation, P4HP will be evaluated in terms of multidisciplinary collaboration, facilitators, and barriers by in-depth interviews with a purposeful sample of midwives, dieticians and pregnant women involved in the C-RCT. Semi-structured interview guides will be prepared for each of the interviews with midwives, dieticians, and pregnant women. 10-15 interviews will be performed with each of the three groups, depending on data saturation, and performed at T1.

### Intervention

P4HP is a non-invasive empowerment intervention and consists of four extra moments for pregnant women to discuss nutrition with their midwife and a dietician. P4HP distinguishes itself from standard birth care by its empowering approach towards improving diet quality during pregnancy. The intervention is free of charge for the women and takes place in an individual or group setting (via CenteringPregnancy). P4HP is designed to be flexible, meaning that the professional has the freedom to adapt to what the individual or group needs at each session. This research is in line with current Dutch policy regarding empowerment, dietary guidelines, and prevention [96–99].

Table 1 provides an overview of the P4HP-elements. For each element of the intervention, it explains the goal(s), the activity, the estimated time that is needed for the activity, and the tools as well as who guides the activity. The exact time investment per session topic will be discussed with and adapted to the possibilities of the midwives and dieticians.

**Table 1:** Overview of the P4HP-intervention

Session	Time	Goal(s)	Activities	Tool
**1. Early nutritional information with midwife (4-10 weeks of participants’ pregnancy)**				
**A) Individual setting**	15 min	Gain insight into perspectives of the women towards nutrition and create a sense of urgency for healthy nutrition during pregnancy Identify aspects that go well regarding nutrition and define at least one achievable step the woman is willing to work on Create a positive attitude towards nutritional changes Understand the complexity of each woman’s situation regarding healthy nutrition	First interview on nutrition	Visual aid to guide the interview and write down action points Motivational Interviewing
**2. Appointment with dietitian (~ 12 weeks of participants’ pregnancy)**				
**A) Individual setting**	30-45 min	Support women in developing strategies to cope with individual challenges Provide more in-depth information	Discuss challenges in healthy nutrition based on the woman’s individual needs	Visual aid to guide the interview and write down action points
**B) Group setting**	60 min	Support women in developing strategies to cope with individual challenges Provide more in-depth information Peer support and learn from each other’s questions and experiences	Workshop with group discussions on challenges in healthy nutrition based on the women’s individual needs Use each other’s experiences to find individual coping mechanisms	Visual aid to guide the interview and write down action points
**3. Reflection moments with midwife (~ 22 and ~ 32 weeks of participants’ pregnancy)**				
**A) Individual setting**	15 min	Increase awareness of the connection between the women’s actions and results on diet quality Increase abilities to identify actionable and achievable goals that lead to improved diet quality	Facilitate women’s reflection on their nutrition and the efforts they made in the last weeks Identify next steps for improvement, if achievable at that moment	
**B) Group setting**	30 min	Increase awareness of the connection between the women’s actions and results on healthy nutrition Increase abilities to identify actionable and achievable goals that lead to improved diet quality Peer support and learn from each other’s questions and experiences	Facilitate women’s reflection on their nutrition and the efforts they made in the last weeks Identify next steps for improvement, if achievable at that moment	

The financial compensation for the invested time by midwives and dieticians will be reimbursed. Participating women will not have additional costs as compared to standard birth care.

### Control

Midwifery practices in control clusters will provide participating women with standard birth care (according to the present Dutch birth care standards [100]) and the usual information on nutrition during pregnancy. There is no standard protocol for nutrition communication in antenatal care, so the amount of time and content can vary between the control practices. It is common practice to dedicate a small amount of time (2-10 min) to the discussion of nutrition at the first consultation, focusing on foods that cannot be safely consumed during pregnancy. In standard birth care, pregnant women may be referred to organizations such as the Netherlands Nutrition Centre for questions, nutritional guidelines, or advice. Frequently used tools such as the app ZwangerHap [101] are likely used by pregnant women in the control group. In addition, the newest dietary guidelines for pregnant women of the Dutch Health Council are freely available for health professionals and pregnant women. Being part of the control group in no way limits the use of such nutritional resources, as they are part of standard birth care. Outcome measures will be obtained in the same way for participants in the control clusters as for those in intervention clusters at baseline (T0) and follow-up (T1).

### Ethical consideration

Ethics approval was given by Medical Research Ethics Committee Utrecht, the Netherlands on September 21st 2021. The committee thereby declares that the proposal satisfactorily deals with ethical issues and that it complies with the Netherlands Code of Conduct for Scientific Practice. Because of negligible risk for participants, the MREC Utrecht has granted this study exemption from the obligation to the insurance that covers damage caused by the research through injury of the participant. Therefore, adverse events are currently not foreseen, due to the nature of the study and intervention. Informed consent will be obtained from each participant, after the purpose and possible consequences of the study have been explained. This study will be conducted according to the principles of the Declaration of Helsinki (October 2013) and according to the Medical Research Involving Human Subjects Act (WMO).

Participation in the study is voluntary and participants can leave the study at any time for any reason if they wish to do so without any consequences. They are asked to inform either the principal investigator or intervention deliverer (midwife or dietician) about their decision. Participants are not obliged to inform the researchers about their reason to withdraw. The investigator can decide to withdraw a participant from the study for urgent medical reasons, such as having a miscarriage or a pregnancy with extreme complications. The reason for withdrawal reason will be kept for the record for further study. Since P4HP is possibly delivered in a group format, there will be no replacement of individual participants after withdrawal.

### Data analyses

#### Quantitative analyses C-RCT

Statistical analyses will be carried out using IBM SPSS Version 25 (Statistical Package for Social Sciences). Data cleaning will be performed before the final review to check for missing data or outliers. We expect data missing will be at random, and if so, all available data from T0 and T1 will be used to conduct the analyses.

All data will be quantitatively presented in tables (Tables 2 and 3). BMI, the DHD2015-index, PRES score, SoC-3 score, QoL VAS score, and GSRH-score will be presented as ordinal data. Ethnicity, education, and living situation will be presented as nominal data. All data will be entered and verified, and scores will be calculated for multiple-item instruments (i.e. DHD2015-index, PRES, SoC-3). Descriptive statistics will be performed to tabulate mean (or median) values of all study characteristics and baseline values of the independent variables. Chi-square (for categorical variables) and Student’s t-tests (for continuous variables) will be used to compare the descriptive statistics between study groups and to identify potential covariates. The number of participants, as well as means (standard deviations, SDs), median or % (numbers of patients), will be tabulated where appropriate.

**Table 2:** Baseline characteristics for intervention and control group

	Intervention group (P4HP)	Control group (standard birth care)	Difference in means (95% CI)	*P*-value
	**Mean (SD)**	**Mean (SD**		
*Age*				
	**% (n)**	**% (n)**		
*Ethnicity (% native Dutch)*				
*Education (low, medium, high)*				
*Living situation (alone, with a partner, with children, with partner and children)*				
	**Mean (SD)**	**Mean (SD)**		
*BMI* *Diet quality (DHD2015-index)*				
*Empowerment (PRES)*				
*Sense of Coherence (SOC-3)*				
*Quality of Life (QoL VAS)*				
*Self-Rated Health (GSRH)*

**Table 3:** Baseline, post-intervention, and change scores for intervention and control group

	T0 *(M, SD)*	T1 *(M, SD)*	Change T1 *(change ± SE)*	Effect estimate *(95% CI)*	Group differences *(P-value)*
BMI *(kg/m2)* Intervention (n=) Control (n=) Effect size					
Diet quality *(DHD2015-index)* Intervention (n=) Control (n=) Effect size					
Empowerment *(PRES)* Intervention (n=) Control (n=) Effect size					
Sense of Coherence *(SOC-3)* Intervention (n=) Control (n=) Effect size					
Quality of Life *(QoL VAS)* Intervention (n=) Control (n=) Effect size					
Self-Rated Health *(GSRH)* Intervention (n=) Control (n=) Effect size					

Linear mixed models will be used to analyse the data. Using linear mixed models allows for the analysis of different sources of variation in data and for unequal variances and correlations. This flexible method is suitable for analysis of the clustered data as it allows to calculate the treatment effect. When there are multiple levels, such as pregnant women seen by the same midwifery practice, the variability in the outcome can be thought of as either within-group or between-group. Pregnant women-level observations are not independent, as within a midwifery practice pregnant women and their guidance are more similar. Units samples at the highest level (in our research, midwifery practices) are regarded as independent. With this method also confounders can be taken into account [102]. After performing the linear mixed models analysis, the final results will be presented in Table 3. This table will display means and standard errors, the between-group differences, and the *p*-values for the treatment effect for all primary and secondary outcomes.

All primary and secondary outcomes will be tested via linear mixed models. Other subgroup analyses include age, ethnicity, individual or group (CenteringPregnancy) consultation, educational level, living situation, working situation, and income level. These variables will be checked if they differ across the groups using an independent Student’s t-test when continuous and normally distributed. If skewed, a Wilcoxon signed-rank test will be done. Variables that are not continuous, will be checked for differences between groups using a chi-square test. Two-sided *p* values < 0.05 will be regarded as statistically significant.

#### Qualitative analyses process evaluation

Stakeholder interviews will be recorded, transcribed verbatim, and analysed in Atlas.ti using inductive coding to derive themes, theories, or concepts from the raw data and to reveal underlying structures of experiences or processes [103]. The coding process will be done by at least two researchers to increase the validity of the process.

## Discussion

This paper describes the study protocol for a mixed methods study consisting of a C-RCT with an intervention group and a control group and a process evaluation. The study protocol includes the evaluation of P4HP, an empowerment intervention to improve diet quality among pregnant women. To our knowledge, this is the first C-RCT that evaluates the effectiveness of an empowerment intervention to improve diet quality in The Netherlands. Research in this field is needed because there is limited evidence of effective empowerment interventions regarding diet quality during pregnancy. To ensure that P4HP fits into standard birth care, various stakeholders have been involved in all steps of the development process. Our study will provide important and unique information on how to empower pregnant women to achieve improved diet quality by midwives and dieticians. Having both a quantitative and qualitative evaluation of P4HP will create a comprehensive overview of both the impact of P4HP and how best to implement it more broadly in practice.

P4HP will be assessed on diet quality, empowerment, SOC, QoL, and SRH. Although these assessments were selected intentionally based on previous research, the intervention may still produce an effect that is not directly assessed by our quantitative assessments. The process evaluation is therefore added to capture these indirect effects using semi-structured interviews. Innovative is that outcome measures include empowerment and SOC, something not common in C-RCT studies. Previous studies found evidence that SOC significantly changed and that those with a weaker SOC were more likely to have a stronger SOC after the intervention that included experimental learning [86, 104], as these groups have most to gain. In case we find change in SOC, it indicates that participants of P4HP benefit from the intervention; providing a more complete picture of the interventions’ successes. Two limiting factors of using Eetscore to assess diet quality are that it is only available in Dutch and oriented to a Dutch dietary pattern. Consequently, this unfortunately limits women who do not speak Dutch and with other diet patterns from participating.

Materials and language used in P4HP are designed to be suitable for low SES pregnant women – the group who will mostly benefit from this intervention because of a general sub-optimal adherence to dietary guidelines [8–10]. We assume P4HP thus aligns with women of all SES groups. As it is not ethical to discriminate the inclusion of participants on their SES-status, all SES-groups visiting participating midwifery practices will be included in this study. In the results we will report on differences in outcomes between SES groups.

This study will make a significant and to our knowledge unique scientific and socially relevant contribution about using an empowerment intervention to improve the diet quality of pregnant women in the Netherlands. If P4HP improves pregnant women’s diet quality, empowerment and other health-related outcomes, the impact may have health, social, and economic benefits. We anticipate that the study outcomes have the potential to change the way nutrition is addressed during pregnancy. The findings will directly benefit pregnant women and their children, as well as inform academics and others who strive to produce interventions that can be effectively implemented in routine care using multisectoral collaboration. If P4HP proves to be an effective and feasible intervention, further research will be done on the extension towards the preconception and postpartum phase.

## Trial status

The cluster randomized trial of P4HP will starts Q1 2022. After implementation and evaluation, the final results will be available by Q4 2023.

## Supplementary Information

**Additional file 1.** Completed SPIRIT guidelines checklist for this P4HP cluster randomized trial protocol article.

## Data Availability

Data sharing does not apply to this article as no datasets have yet been generated or analysed.

## Abbreviations

C-RCT: Cluster randomized controlled trial
DHD2015- index: The Dutch Healthy Diet index 2015
GSRH: General Self-Rated Health
P4HP: Power 4 a Healthy Pregnancy
PRES: Pregnancy Related Empowerment Score
QoL: Quality of Life
SES: Socioeconomic status
SOC: Sense of Coherence
SRH: Self-Rated Health
SPIRIT: Standard Protocol Items Recommendations for Interventional Trials
VAS: Visual Analogue Scale
