# Correction to: Evaluating ‘Power 4 a Healthy Pregnancy’ (P4HP) – protocol for a cluster randomized controlled trial and process evaluation to empower pregnant women towards improved diet quality

**DOI:** 10.1186/s12889-022-12714-y

**Published:** 2022-02-14

**Authors:** Renske M. van Lonkhuijzen, Susanne Cremers, Jeanne H. M. de Vries, Edith J. M. Feskens, Annemarie Wagemakers

**Affiliations:** 1 Health and Society, Department of Social Sciences, Wageningen University & Research, Hollandseweg 1, bode 60, 6706KN Wageningen, The Netherlands; 2 Human Nutrition & Health, Department of Agrotechnology and Food Sciences, Wageningen University & Research, Stippeneng 4, bode 62, 6708WE Wageningen, The Netherlands

**Correction to: BMC Public Health 22, 148 (2022)**

**https://doi.org/10.1186/s12889-022-12543-z**

The original publication of this article [1] contained several errors in the author names. The incorrect and correct author names are listed in this correction article. The original article has been updated.

**Incorrect author names**

- R. M. Renske van Lonkhuijzen
- S. Susanne Cremers
- J. H. M. Jeanne de Vries
- E. J. M. Edith Feskens
- M. A. E. Annemarie Wagemakers

**Correct author names**

- Renske M. van Lonkhuijzen
- Susanne Cremers
- Jeanne H.M. de Vries
- Edith J.M. Feskens
- Annemarie Wagemakers

## References

[1] van Lonkhuijzen RM (2022). Evaluating ‘Power 4 a Healthy Pregnancy’ (P4HP) - protocol for a cluster randomized controlled trial and process evaluation to empower pregnant women towards improved diet quality. BMC Public Health.

